# Temperature Studies with the Asian Citrus Psyllid, *Diaphorina citri*: Cold Hardiness and Temperature Thresholds for Oviposition

**DOI:** 10.1673/031.011.8301

**Published:** 2011-07-02

**Authors:** David G. Hall, Erik J. Wenninger, Matthew G. Hentz

**Affiliations:** ^1^ USDA-ARS, 2100 South Rock Road, Fort Pierce, FL, USA; ^2^ Department of Plant, Soil, and Entomological Sciences, University of Idaho, Twin Falls Research and Extension Center, Twin Falls, ID 83303-1827, USA

**Keywords:** insecticide, cold acclimation, *Candidatus* Liberibacter asiaticus, Huanglongbing

## Abstract

This study was conducted to obtain information on the cold hardiness of the Asian citrus psyllid, *Diaphorina citri* Kuwayama (Hemiptera: Psyllidae), in Florida and to assess upper and lower temperature thresholds for oviposition. The psyllid is an important pest in citrus because it transmits the bacterial pathogens responsible for citrus greening disease, Huanglongbing, considered the most serious citrus disease worldwide. *D. citri* was first found in Florida during 1998, and the disease was discovered during 2005. Little was known regarding cold hardiness of *D. citri,* but Florida citrus is occasionally subjected to notable freeze events. Temperature and duration were each significant sources of variation in percent mortality of *D. citri* subjected to freeze events. Relatively large percentages of adults and nymphs survived after being exposed for several hours to temperatures as low as -5 to -6° C. Relatively large percentages of eggs hatched after being exposed for several hours to temperatures as low as -8° C. Research results indicated that adult *D. citri* become cold acclimated during the winter through exposure to cooler winter temperatures. There was no evidence that eggs became cold acclimated during winter. Cold acclimation in nymphs was not investigated. Research with adult *D. citri* from laboratory and greenhouse colonies revealed that mild to moderate freeze events were usually nonlethal to the *D. citri* irrespective of whether they were cold acclimated or not. Upper and lower temperature thresholds for oviposition were investigated because such information may be valuable in explaining the geographic distribution and potential spread of the pest from Florida as well as how cooler winter temperatures might limit population growth. The estimated lower and upper thresholds for oviposition were 16.0 and 41.6° C, respectively; the estimated temperature of peak oviposition over a 48 h period was 29.6° C.

## Introduction

The Asian citrus psyllid, *Diaphorina citri* Kuwayama (Hemiptera: Psyllidae), is an important citrus pest because it transmits phloem-limited bacteria responsible for citrus greening disease (Huanglongbing), regarded as the world's most serious disease of citrus (McClean and Schwartz 1970; Halbert and Manjunath 2004; Bové 2006). *D. citri* has been known in Florida since June 1998 (Tsai et al. 2002) and is established throughout the state's citrus-growing regions (Michaud 2004). Subsequently, *D. citri* has spread across the southern areas of the United States — Arizona, Alabama, California, Georgia, Louisiana, Mississippi, South Carolina, and Texas. Citrus greening attributed to *Candidatus* Liberibacter asiaticus (Rhizobiales: Rhizobiaceae), has been known in Florida since August 2005 and is widespread in Florida citrus (FDACS 2006). The disease has also now been found in Georgia, Louisiana, and South Carolina.

Information is available on the effects of temperature on the biology of *D. citri* as it relates to the development of immatures and longevity of adults (Fung and Chen 2006; Liu and Tsai 2000; Nakata 2006; Nava et al. 2007; Pande 1971). However, little information has been published on the cold hardiness of *D. citri.* Diapause can afford some degree of cold protection for insects during the winter, but no reports were found of *D. citri* undergoing a winter diapause. *D. citri* inhabits tropical or subtropical areas where winter temperatures are usually mild. Adults pass the winter months on their host plants feeding on mature leaves. Ashihara (2004) reported survival rates of 4–6% of a population of *D. citri* in Kuchinotsu (northern Kyushu, Japan) following a winter with a minimum temperature of -3.3° C. Mizuno et al. (2004) reported 63 or 100% mortality of adult *D. citri* following two or four days, respectively, of exposure to 0° C with no significant difference in mortality rates of males and females.

Florida citrus is periodically subjected to freezes — particularly citrus grown in the central and north central areas of the state (Miller and Glantz 1988). During 1766–1989, the Florida citrus industry suffered 23 freeze events of each which caused so much damage that a number of years were required for trees to recover (Rogers and Rohli 1991). *D. citri* has spread throughout the state including to the north of the citrus industry on other host plants such as orange jasmine, *Murraya paniculata* (L.) Jack (Sapindales: Rutaceae). A hard or severe freeze event in a northern area might eliminate *D. citri* for some period of time until new individuals migrate to repopulate the area. The northern spread of *D. citri* in the United States would be contingent on its ability to withstand freezes. Some freeze events may occur that are nonlethal to *D. citri,* thus it is important to understand what temperatures *D. citri* can survive.

Liu and Tsai (2000) and Fung and Chen (2006) reported on differences in oviposition rates by *D. citri* at different temperatures, but did not specifically investigate upper and lower temperature thresholds for oviposition. Information on these thresholds would be valuable in developing population models for *D. citri* and for explaining the geographic distribution and potential spread of *D. citri.* Temperature thresholds for oviposition have been used to forecast the geographical spread of other insects such as *Diaprepes abbreviatus,* an important weevil pest of citrus in Florida, Texas, and California (Lapointe et al. 2007).

The objectives of this study were (1) to investigate mortality rates of *D. citri* nymphs and adults, and percent hatch of eggs, following exposure of these life stages to different freeze events varying in intensity and duration but all of which could realistically occur in Florida; and (2) to assess upper and lower temperature thresholds for oviposition activity by female *D. citri.*

## Materials and Methods

The insects for these studies were obtained from a colony established during early 2000 at the USDA-ARS U.S. Horticultural Research Laboratory, Fort Pierce, FL. Originally collected from citrus, *D. citri* have since been continuously reared in Plexiglas (0.6 × 0.6 × 0.6 m) or BugDorm—2 cages (MegaView, Science Education Services Co., Ltd., www.megaview.com) containing orange jasmine, *M. paniculata* (Hall et al. 2007). The colony is located in a small temperature-controlled (air conditioned/heated) greenhouse and maintained by adding new *M. paniculata* plants into cages on a 2—week schedule (Skelley and Hoy 2004) with no introduction of wild types. Care is taken to keep the citrus greening pathogen, *C.* L. asiaticus, from being introduced into the colony, and samples of *D. citri* from the colony are thus monitored for the pathogen once every three months (Li et al. 2006). The mean (±SEM) temperature in the air-conditioned greenhouse during the course of these studies was 27.8 ± 0.3° C. The mean (±SEM) minimum daily temperature in the temperature controlled greenhouse was 24.9 ± 0.4° C. In addition to the greenhouse colony, adults from the greenhouse colony were used to establish a colony during fall 2008 in an environmental chamber held at 25.8 ± 0.8° C, and *D. citri* from this colony were used in studies on cold acclimation.

The temperature studies were conducted using either a low-temperature version of the Percival I36LL environmental chamber (for research on mortality of *D. citri* subjected to freezes) or a conventional Percival I36LL environmental chamber (for research on temperature thresholds for oviposition) (both chambers by Percival Scientific, Inc., www.percival-scientific.com). For research on mortality of *D. citri* exposed to freezes, two temperature loggers (WatchDog Model 250, Spectrum Technologies, Inc., www.specmeters.com) (accuracy ± 0.06° C) were placed into the chamber to monitor temperatures (temperature recordings every 12 min). The same temperature logger was used throughout these experiments.

### Cold hardiness

**Experiment 1: Adults from a temperaturecontrolled greenhouse.** Mortality of adult *D. citri* was investigated following exposure to target freezes of -1.5, -2.5, -3.5, -4.5, -5.0, -5.5, -6.5, -7.5, -8.5, or -9.5° C at different hourly durations of from 1 to 10 h. A group of five males and a group of five females (all adults 7–10 d old) from the greenhouse colony was placed into a pre-chilled Petri dish (suspension culture dish, 35mm × 10mm, non-treated polystyrene, #430588, Corning Inc., www.corning.com/lifesciences) and held at a specific temperature for a specific period of time. Similar groups of males and females not exposed to cold served as control groups. After each exposure period, each group of adults was transferred to a Petri dish containing a fresh citrus leaf disk (described below) imbedded on agarose (7 g/500 ml water), and the groups of adults on leaf disks were then held at 26° C (within the optimum temperature range for *D. citri,* Fung and Chen 2006) in an environmental chamber (14:10 L:D). Each group of adults was examined at the end of a freeze event and thereafter daily for seven days to determine how many adults survived. There were two replicate groups of each sex for each exposure period to each temperature for a total of 1010 *D. citri* of each sex including controls. The experiment was conducted over time randomly testing one temperature at a time. All adults tested at a given temperature were placed into the chamber at the same time, and those assigned to each exposure period were removed after the appropriate amount of time. This required briefly opening the chamber door for ∼5 sec every hour. Mean percent mortality (corrected for mortality of controls) was computed daily for each group of male or female *D. citri* following each freeze event. A contour plot was used to summarize adult mortality at different temperatures and freeze durations. An analysis of variance was conducted to assess the significance of temperature, duration, day after a freeze, and sex on percent adult mortality. A probit analysis was conducted on percent mortality data associated with each temperature to estimate freeze durations required to kill 50 (LT_50_) or 95 (LT_95_) % of *D. citri.*

Leaf disks for the study were prepared by taking a whole citrus (‘Duncan’ grapefruit, *Citrus paradisi* Macfadyen (Sapindales: Rutaceae)) leaf and rinsing it in cold tap water; it was then surface sterilized by soaking in a 5% bleach solution for 30 – 40 sec and rinsed several times with tap water again, and then allowed to air-dry. A leaf disk was then cut by boring through a leaf with a 2.34 cm diameter copper pipe with sharpened edges. The pipe was sterilized by first washing it with antibacterial soap, then rinsing it several times with tap water, and then submersing it in 70% ethanol for 60 sec, after which it was allowed to air dry.

The basic procedures used for this experiment with adults from the temperature-controlled greenhouse were used in the following experiments on cold hardiness.

**Experiment 2: Adults from indoor and outdoor colonies during winter.** Cold acclimation by adult *D. citriwas* investigated by maintaining a cage of *D. citri* on *M. paniculata* outdoors during the winter (December — March). *D. citri* from the temperature-controlled greenhouse colony were used to establish this outdoor colony on 1 December 2008. On 16 January 2009, ten males and ten females from both the outdoor and chamber colonies were held at a target temperature of-7.0° C for 4, 5, or 6 h. Similar groups from the two colonies of males and females not exposed to cold served as control groups. After being subjected to the freeze events, each group of *D. citri* was examined four days later to determine how many adults survived. For *D. citri* from the outdoor colony as well as *D. citri* from the chamber, there were two replicate groups of each sex for each exposure period for a total of 160 adults (including controls) from each colony. The experiment was repeated on 10 March 2009 and again on 17 March. Air temperatures out of doors during these winter tests were obtained from a data logger inside the outdoor cage (hourly records).

The 4, 5, and 6 h exposure periods used in this experiment were chosen based on the results of the first experiment, which indicated that these exposure times to -7.0° C were long enough to kill a large percentage of adults; it was hypothesized that if adults from the outdoor colony were cold acclimated that lower percentages of these adults would die after a 4 to 6 h freeze than those from the chamber colony.

Mean percent mortality (corrected for control mortality) was computed for each colony group of male or female *D. citri* following each freeze event. An analysis of variance was conducted to assess the significance of freeze duration, colony, and sex on percent adult mortality. Mean minimum outdoor daily air temperatures were calculated for a two-week period prior to each of the experiments. Correlation analyses (Pearson's correlation coefficient) were conducted to determine if there were significant relationships between percent *D. citri* mortality and these minimum air temperatures.

**Experiment 3: Eggs from a temperaturecontrolled greenhouse.** Percent hatch of *D. citri* eggs was investigated following exposure to target freezes of-1.5, -2.5, -3.5, -4.5, -5.0, -5.5, -6.5, -7.5, or -8.5° C at different hourly durations of from 1 to 10 h. Young *M. paniculata* shoots with newly deposited *D. citri* eggs (<24 h old) from the greenhouse colony were placed into small Petri dishes (one shoot per dish) and held at a temperature for the duration of the freeze. The number of eggs on each shoot was counted under a microscope prior to exposing the eggs to cold. After each exposure period, each shoot with eggs was transferred to a leaf disk. The shoots were examined under a microscope 5 d later to determine the number of eggs that hatched. There were three shoots with eggs exposed to each temperature for each exposure period. For each temperature, numbers of eggs that hatched were also determined for three egg-infested shoots that were not exposed to cold (control treatment). Overall, there was a mean (±SEM) of 30.8 ±0.9 eggs per shoot in this study. Mean percentage of eggs that did not hatch (corrected for the percentage of control eggs that did not hatch) was computed for each group of eggs for each freeze event. A probit analysis was conducted on percentages of eggs that failed to hatch following exposure to each temperature to estimate LT50 and LT95 values.

**Experiment 4: Eggs from an outdoor colony during the winter.** The cold hardiness of *D. citri* eggs during the winter was investigated. On 26 March 2009, young *M. paniculata* shoots with newly deposited eggs were obtained from the outdoor colony. These were placed individually into small Petri dishes and held at a target temperature of-7.0° C for 1, 2, 3, 4, 5, 6, 7, or 8 h. Simultaneously, shoots with newly deposited eggs from the chamber colony of *D. citri* were subjected to the same freeze events for comparisons. The shoots were examined 5 d later to determine the number of eggs that hatched. There were three shoots with eggs exposed to each temperature for each exposure period [mean (±SEM) of 28.6 ± 1.8 eggs per shoot]. For each colony, numbers of eggs that hatched were also determined for three egg-infested shoots that were not exposed to cold (control treatment). Paired *t-*tests were conducted for each freeze duration and overall durations to compare eggs from the chamber and outdoor colonies with respect to percentages that did not hatch (corrected for control percentages).

**Experiment 5: Nymphs from a temperature — controlled greenhouse** Mortality of *D. citri* nymphs was investigated following exposure to target freezes of -1.5, -2.5, -3.5, -4.5, -5.5, -6.5, -7.5, or -8.5° C at different hourly durations of from 1 to 10 h. Young *M. paniculata* shoots with fifth-instar *D. citri* nymphs from the greenhouse colony were placed into pre-chilled Petri dishes (one shoot per dish) and held at a temperature for the duration of the freeze. The number of nymphs on each shoot was counted prior to exposing the nymphs to cold. After each exposure period, nymphs were removed with a camel hair paint brush and transferred to a leaf disk. Each leaf disk was examined 24 h later to determine the number of nymphs that died. Three shoots with nymphs were exposed to each temperature for each exposure period [a mean (±SEM) of 26.6 ± 0.7 nymphs per flush shoot]. For each temperature, the numbers of nymphs that died were determined for three shoots with nymphs that were not exposed to cold (control treatment). Mean percent mortality of nymphs (corrected for mortality of controls) was computed for each freeze event. A probit analysis was conducted on percent nymph mortality following exposure to each temperature to estimate LT_50_ and LT_95_ values.

### Statistical analyses and data evaluation

Percent mortality or hatch data from insects subjected to each freeze event were corrected for data from *D. citri* not exposed to the freeze events using the formula by Abbott (1925). Color-filled, contour spectrum plots were generated using SigmaPlot (Systat Software 2008) to summarize the three dimensional data (freeze temperature, freeze duration, and percent mortality or hatch). Regression analyses were conducted to relate percent mortality or hatch (Y) to freeze event temperature (X_1_) and duration (X_2_) using PROC GLM (SAS Institute 2008). Correlation analyses were conducted using PROC CORR (SAS Institute 2008). Analyses of variance on percent mortality or hatch data were conducted using either PROC ANOVA (SAS 2008) or PROC TTEST (SAS Institute 2008) on arcsine-transformed percentages. For eggs or nymphs, statistical analyses were weighted on the initial number of live eggs or nymphs per shoot. Probit analyses were conducted using PROC PROBIT (SAS Institute 2008).

### Temperature thresholds for oviposition

All *D. citri* used for oviposition experiments were observed on ‘Duncan’ grapefruit seedlings caged in plastic vial containers (Wenninger and Hall 2007). Each cage consisted of a 52 mm-tall vial, modified as an open-ended cylinder with a foam plug used to stopper the top opening and two ventilation holes on the sides; individual cages were slipped over a seedling grown in a coneshaped planting container. *D. citri* were held in an environmental chamber at 26° C, 60% RH, and 14:10 L:D photoperiod, which resulted in 70–80% RH inside the vials. Adult *D. citri* approximately 7 d of age were collected from the greenhouse colony. Female/male pairs were established on seedlings in the vial containers, and after 2–3 d each seedling was checked for the presence of eggs. Males were removed from vials in which females had laid eggs, and females that had not laid eggs were discarded. Females that laid eggs were assumed to be mated, which was later confirmed by observing the emergence of nymphs on each seedling. Mated females were transferred individually to new grapefruit seedlings, and were moved to an environmental chamber maintained at 60% RH, 14:10 L:D photoperiod, and one of 11 temperatures: 11, 14, 17, 20, 23, 26, 29, 32, 35, 38, or 41° C (N = 10 females per temperature treatment). After 48 h each female was removed from her vial, and the number of eggs laid was counted. The number of eggs laid per female was analyzed by both linear and quadratic regression to estimate the lower and upper thresholds for oviposition as well as the temperature of peak oviposition. Because none of the females in the 11 or 14° C treatments laid eggs, they were excluded from regression analyses. Immediately following the termination of the 11° C treatment, these females were transferred to a 26°C chamber for an additional 48 h to assess oviposition. Females in the 14° C treatment were not further evaluated.

## Results

### Cold hardiness

**Experiment 1: Adults from a temperaturecontrolled greenhouse.** Mean (±SEM) temperatures recorded during the freeze experiments were close to targeted temperatures: -1.5 ± 0.02, -2.6 ± 0.02, -3.6 ± 0.02, -4.5 ± 0.03, -5.0 ± 0.04, -5.5 ± 0.03, -6.5 ± 0.03, -7.4 ± 0.03,-8.5 ± 0.03, and -9.2 ± 0.08° C. Few adults died when exposed to temperatures of -4.5 or colder for only several hours, but more died when exposed to these temperatures for longer periods of time. Some freeze events were lethal to *D. citri,* but death occurred slowly over 3–4 days with little or no additional mortality occurring afterwards. Four days was therefore used as the time to assess adult mortality following all freeze events.

**Table 1.**
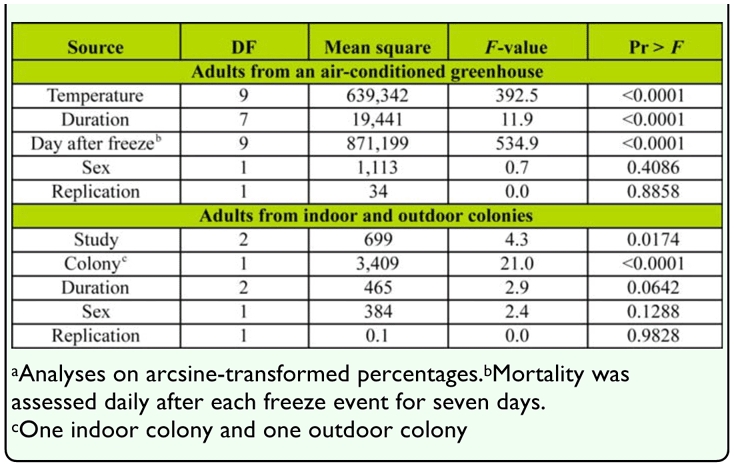
Results of analyses of variance on percent mortality of adult *Diaphorina citri* exposed to various freeze events.^a^

**Figure 1.**
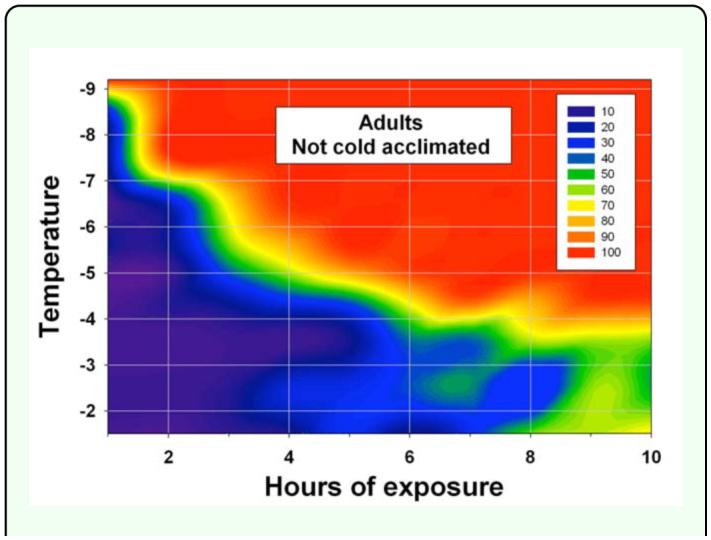
Percent mortality of adult *Diaphorina citri* after being exposed to from -1.5 to -9.2 °C for one to ten hours (two replications of five adults of each sex for each temperature/exposure combination, data pooled over both sexes and replications). The adults were from an air-conditioned greenhouse and, thus, not cold acclimated. This figure identifies freeze events that should usually be nonlethal to the adult psyllids irrespective of whether they are cold acclimated. High quality figures are available online.

**Figure 2.**
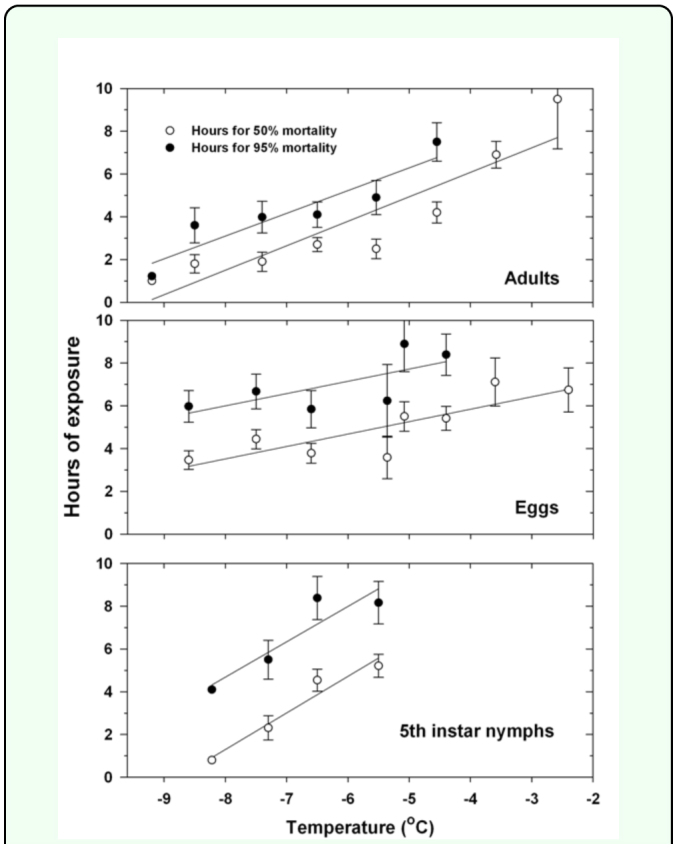
Results of probit analyses on the duration of a freeze required for 50 or 95% mortality of *Diaphorina citri* adults and nymphs and for reduction levels of 50 or 95% in egg hatch. The insects were from an air-conditioned greenhouse and thus not cold-acclimated. High quality figures are available online

Temperature and duration were each significant sources of variation in percent mortality of adults subjected to freeze events while sex was not (Table 1). Percent mortality of adult *D. citri* generally increased as the temperature decreased and/or the freeze duration increased (Figure 1). Few adults generally died when they were exposed for 1– 2 h to temperatures of -5.0° C or colder, but many died when they were exposed to these temperatures for four or more hours. Percent mortality of adults (Y) (over both sexes) was statistically related to a freeze event's temperature (X_1_) and duration in hours (X_2_): Y = -10.6X_1_ + 7.5X_2_ -34.2 (*F* = 2,860; Pr > *F* = < 0.0001; R^2^ = 0.64; 3,199 d.f). The lethal time of exposure for 95% mortality (LT_95_) of adults ranged from about 7 h at -4.5° C to under 2 hr at -9.2° C (Figure 2). As temperatures increased from -8.5 to -4.5° C, 1–2 h more exposure time was required for 95% mortality as compared to 50% mortality. Ten hours of exposure at temperatures of — 1.47, -2.58, or -3.58° C resulted in less than 95% mortality.

**Experiment 2: Adults from indoor and outdoor colonies during winter.** Study and *D. citri* colony (indoor or outdoor) were each significant sources of variation in percent mortality while sex and exposure time were not (Table 1). Based on minimum air temperatures during the two weeks preceding each study, the outdoor colony of *D. citri* was exposed to the coldest weather prior to the second study. The mean (±SEM) minimum air temperature prior to the first, second, and third studies was 11.6 ± 0.7, 9.7 ± 0.7, and 11.9 ± 1.0° C, respectively. Greater percentages of adults from the indoor chamber colony than the outdoor colony died during the second (coldest) study (Table 2). Mean percent mortality of adult *D. citri* from the outdoor colony was positively correlated with mean minimum daily air temperatures during the two weeks prior to the freeze events (r = 0.47, Pr > |r| = 0.004, N = 36). There was no significant correlation between percent mortality of the indoor colony of adults and minimum daily temperatures preceding the studies (r = -0.09, Pr > |r| = 0.59, N = 36).

**Table 2.**
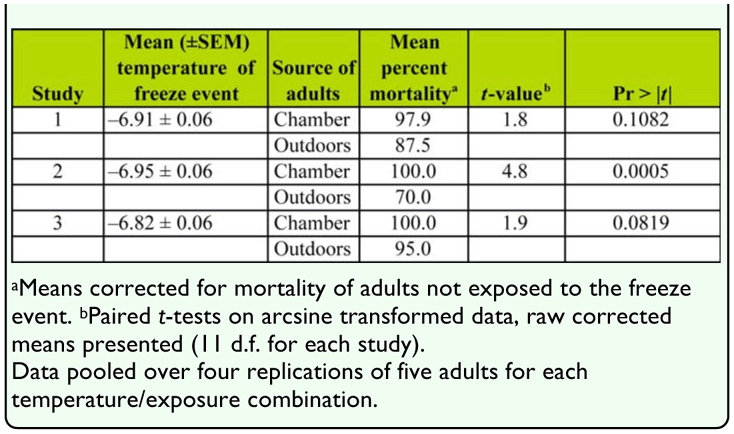
Percent mortality of adult *Diaphorina citri* after a freeze of approximately -7°C for 4 to 6 hours, comparison of adults from a colony in an environmental chamber (25.8 ± 0.8°C) to adults from an outdoor colony during the winter.

**Figure 3.**
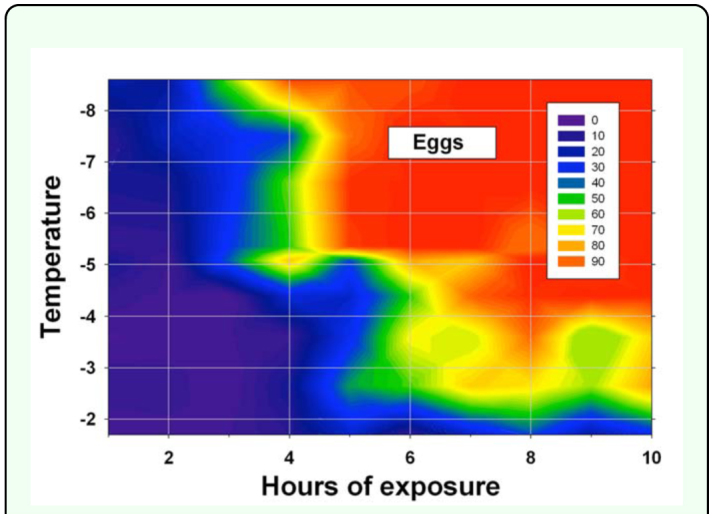
Percentages of egg-stage *Diaphorina citri* that did not hatch after being exposed to freezing temperatures of from -1.7 to -8.6 °C for I to 10 h (data pooled over three egg-infested flush shoots for each temperature/exposure combination, an average of 31 eggs per shoot). High quality figures are available online

**Experiment 3:** Eggs **from a temperaturecontrolled greenhouse.** Temperatures recorded during experiments with eggs were close to targeted temperatures: mean (±SEM) temperatures were -1.7 ± 0.002, -2.4 ± 0.02, -3.6 ± 0.02, -4.4 ± 0.02, -5.1 ± 0.02, -5.4 ± 0.03, -6.6 ± 0.02, -7.5 ± 0.02, and -8.6 ± 0.02° C. Percentages of eggs that did not hatch generally increased as the temperature decreased below -3° C and the duration of the freeze increased to 6 h or longer (Figure 3). The lethal time of exposure for a 95% reduction in egg hatch ranged from about 8 h at -4.5° C to about 6 h at -8.6° C (Figure 2). As temperatures increased from -8.6 to -4.5° C, about twice the exposure time was required for 95% reductions in egg hatch as compared for 50% reductions. Fewer than 90% eggs did not hatch after 10 h of exposure to -2.4 or -3.6° C, and fewer than 50% eggs did not hatch following 10 h exposure to -1.7° C. The following freeze events were projected to result in 0% egg hatch: -4.4° C for at least 7 h; -5.1° C for at least 6 h; and -6.6, -7.5, or 8.6° C for at least 5 h. Percent of eggs that did not hatch (Y) was statistically related to a freeze event's temperature (X_1_) and duration in hours (X_2_): Y = -8.0X_1_ + 10.4X_2_ -44.0 (*F* = 324; Pr > *F* = < 0.0001; R^2^ = 0.71; 263 d.f).

**Experiment 4: Eggs from an outdoor colony during the winter.** The mean (±SEM) temperature recorded during the experiment was -6.99 ± 0.04° C. Percentage hatch of eggs from the environmental chamber did not differ significantly from percent hatch of eggs from the outdoor colony when eggs were exposed to this temperature for 1 to 8 h (hourly data analyses not presented). Over all freeze durations, the mean (±SEM) percentage of eggs that did not hatch was 63.9 ± 8.5% for eggs from the indoor colony and 50.2 ± 9.4% for eggs from the outdoor colony. There was no significant difference between these means (*t* = 0.91, Pr > |*t*| = 0.37, 20 d.f.).

**Figure 4.**
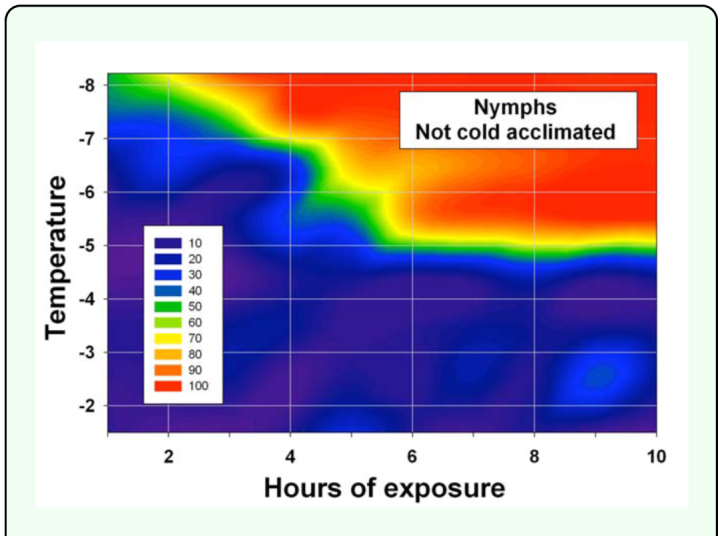
Percent mortality of fifth instar *Diaphorina citri* nymphs after being exposed to -1.5 to -8.2 °C for 1 to 10 h (data pooled over three nymph-infested flush shoots for each temperature/ exposure combination, an average of 27 nymphs per shoot). The nymphs were from an air-conditioned greenhouse and, thus, not cold acclimated. This figure identifies freeze events that should usually be nonlethal to nymphs irrespective of whether nymphs are cold acclimated. High quality figures are available online.

**Experiment 5: Nymphs from a temperature-controlled greenhouse.** Mean (±SEM) temperatures recorded during the experiments were close to targeted temperatures: -1.5 ± 0.01, -2.7 ± 0.01, -3.5 ± 0.02, -4.4 ± 0.02, -5.5 ± 0.02, -6.5 ± 0.02, -7.3 ± 0.02, and -8.2 ± 0.02° C. Up to 10 h of exposure to -1.5, -2.7, -3.4, or -4.4° C resulted in less than 95% mortality of nymphs (Figure 4). The lethal time of exposure for 95% mortality of nymphs ranged from about 8 h at -5.5° C to about 4 h at -8.2° C (Figure 2). As temperatures increased from -8.2 to -5.5° C, 3–4 more hours of exposure time was required for 95% mortality as compared for 50% mortality. Percent mortality of nymphs (Y) was statistically related to a freeze event's temperature (X_1_) and duration in hours (X_2_): Y =-9.7X_1_ + 3.2X_2_ -31.5 (*F* = 32; Pr > *F* = < 0.0001; R^2^ = 0.30; 149 d.f).

### Temperature thresholds for oviposition

**Figure 5.**
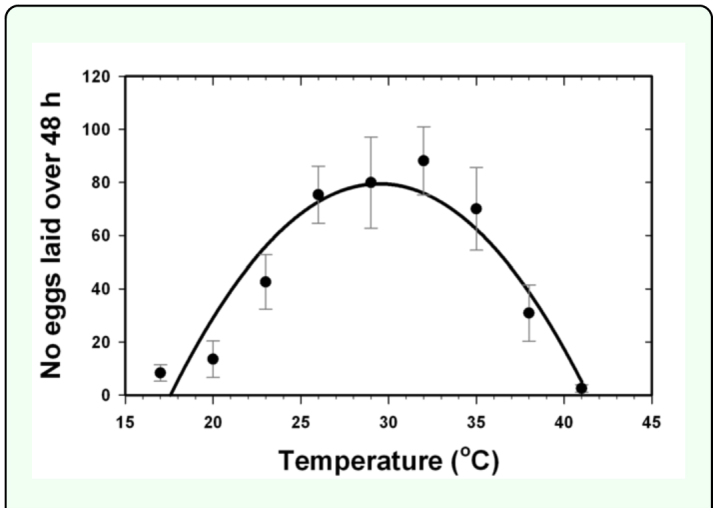
Quadratic relationship between the number of eggs laid by female *Diaphorina citri* over 48 h and temperatures at which females were held. High quality figures are available online.

The number of eggs laid by females (Y) increased linearly with increasing temperature (X) between 17 and 32° C (Y = 6.0X - 95.9, *F* = 46.6, *P* <0.0001, r^2^ = 0.45, 1, 59 d.f). Extrapolation of the linear regression yielded an x-intercept (estimated lower threshold for oviposition) at 16.0° C. Evaluation of the oviposition data between 17 and 41° C showed a significant fit to a quadratic model (Y = 0.6X^2^ + 33.1X - 407.9, *F* = 32.1, *P* <0.0001, r^2^ = 0.42, 2, 89 d.f.; Figure 5). Extrapolation of the quadratic regression yielded x-intercepts at 17.5° C (lower threshold for oviposition) and 41.6° C (upper threshold for oviposition). The estimated temperature of peak oviposition (vertex of the parabola) was 29.6° C.

No oviposition was observed by *D. citri* when held at 11 or 14° C. However, *D. citri* in the 11° C treatment resumed oviposition of fertile eggs following transfer to a 26° C chamber. The mean (±SEM) number of eggs oviposited by these females, 54.3 ± 12.4, did not differ significantly from the number laid by females in the 26° C treatment, 75.3 ± 10.8 (*t* = 1.3, *P* = 0.217, 9 d.f).

Increased mortality was observed in the two highest temperature treatments. Four of the females in the 38° C treatment, and all ten females in the 41° C treatment, died within the 48 h observation period; only one other female among the other treatments died (in the 23° C treatment).

## Discussion

### Cold hardiness

The probability of a freeze at ≤-2.2, ≤ -4.4, or ≤ -6.7° C occurring during the winter in north central Florida was estimated to be 82.4, 47.1, or 17.6%, respectively (Miller and Glantz 1988). Freeze probabilities for Florida citrus declined southwardly. The probability of a freeze at ≤-22, < -4.4, or ≤ -6.7° C occurring in south central Florida was estimated to be 23.1, 7.7, or 0.0%, respectively (Miller and Glantz 1988). Freezes that have affected citrus in Florida have been described as ‘light’ (0 to -2.0° C), ‘moderate’ (-2.0 to -4.0° C), ‘hard’ (4.0 to -6.5° C), and ‘severe’ (< -6.5° C), with ‘hard’ and ‘severe’ freezes causing substantial damage to citrus (Rogers and Rohli 1991). No published information was found on the duration of freeze events experienced in Florida, however, our data suggests that a large majority of *D. citri* would survive the freezes described as ‘light’ or ‘moderate’. Depending on freeze duration and cold acclimation, moderate numbers of *D. citri* might survive a ‘hard’ freeze and even a ‘severe’ freeze. According to data obtained from the Florida Automated Weather Network (http://fawn.ifas.ufl.edu/), a freeze lasting over 10 h occurred during 21–22 January 2009 in east central Florida. The mean (±SEM) temperature during the freeze was -1.3 ± 0.1° C with a period just over 4 h of -1.8 ± 0. 1° C. Based on the results of our study, this freeze event would have caused little direct mortality of *D. citri.*

No information was available on the behavior of adult *D. citri* at the onset of cold weather. Particularly in older trees, adults might defend themselves against a freeze by seeking cold-protected sites on leaves deeper in a tree canopy, possibly on limbs or the trunk of a tree, or they might move to leaf litter on the ground. *D. citri* overwinter as adults, but no winter diapause has been reported for the species. Under winter conditions in Florida, there are sometimes prolonged cold spells during which adults can be found on mature leaves. Occasionally following such cold spells, temperatures increase and trees produce an off-season flush. Under these circumstances in Florida, overwintering adults readily mate and oviposit on the flush. If adult *D. citri* had a winter diapause state, it seems doubtful that adults could respond so quickly to an off-season flush. Adult pear psylla, *Cacopsylla pyricola* (Foerster), overwinter in diapause until late winter or early spring as temperatures warm (Horton et al. 1996). Diapausing *C. pyricola* have been reported to survive after 24 h of exposure to temperatures as low as -18.0° C (Horton et al. 1996).

The ability of an organism to survive exposure to cold temperatures has been referred to as cold hardiness (Lee 1991). Many insects can improve their cold tolerance through a combination of both rapid and longer-term hardening responses (Salt 1961; Denlinger and Lee 1998; Kelty and Lee 2001). Therefore, we choose to compare mortality rates of laboratory *D. citri* to mortality of *D. citri* held in an outdoor enclosure, the latter of which would be subject to field-relevant hardening. This comparison showed that adult *D. citri* become cold-acclimated during the winter through exposure to cooler winter temperatures. Cold acclimation has been demonstrated for a number of insect species, for example the aphid *Sitobion avenae* F. (Powell and Bale 2005); the migratory locust *Locusta migratoria* (Wang and Kang 2003); the fruit fly *Drosophila melangaster* (Yi et al. 2007); and the midge *Belgica antarctdica* (Lee et al. 2006). At any time during the winter, the degree to which adult *D. citri* are cold-acclimated will depend on daily temperatures preceding a freeze event, with greater cold acclimation when temperatures have been cooler. Our data showed that cold tolerance of adult *D. citri* was correlated with temperatures during the weeks preceding a freeze. There was no evidence that eggs from outdoors in the winter were any less cold susceptible than eggs from indoors. Cold acclimation in nymphs was not investigated, but nymphs of other species are known to acclimate to cold — for example nymphs of the aphid *Sitobion avenae* (Powell and Bale 2005) and of the locust *L. migratoria* (Wang and Kang 2003). A freeze event severe enough to kill flush leaves (on which *D. citri* oviposition and nymph development are dependent) would likely result in mass mortality of immatures, except perhaps of older nymphs that might complete development on stems or mature leaves. New citrus flush shoots (5–10 cm length) have been noted as being fairly tolerant of a 3 h freeze at -1.7° C or of a 30 min freeze at -2.2 ° C, but large percentages of new shoots may die following freezes of -2.2° C for 2 to 3 h or following a -3.3° C freeze for 30 min (Oswalt 2008).

### Temperature thresholds for oviposition

Fung and Chen (2006) reported that female *D. citri* lived as long as 300 d at 16° C, but did not lay eggs over that time period, which is consistent with our linear regression-based estimate of 16.0° C for the lower threshold for oviposition. However, Liu and Tsai (2000) reported that females held at 15° C did lay eggs, though the number of eggs laid was less than 25% of the total lifetime number of eggs laid by females held at 28° C. It may be that *D. citri* from different populations exhibit different life history adaptations. For example, Liu and Tsai (2000) reported a minimum temperature threshold for development that was ca. 3° C lower than the value reported by Fung and Chen (2006). It remains to be investigated whether females that are cold-acclimated might oviposit at temperatures below 16.0° C.

Female *D. citri* have been reported to lay more eggs over their lives when held at 28° C versus either 30° C (Liu and Tsai 2000) or 32° C (Fung and Chen 2006). Our estimate of 29.6° C for the temperature of peak oviposition over 48 h is more or less consistent with these values. It might be that females can temporarily increase oviposition during a short exposure to warmer temperatures, but would show higher lifetime fecundity at more moderate temperatures. Oviposition rates in *D. citri* at temperatures above 33° C have not been examined, but Liu and Tsai (2000) and Fung and Chen (2006) showed dramatically reduced oviposition at 33 and 32° C, respectively, and Skelley and Hoy (2004) reported complete cessation of oviposition during a five day period at 34° C. Our results show that females are still able to lay some eggs at least up to 41° C, but these females also suffered high mortality. It is probable that *D. citri* acclimates to heat, as the *D. citri* has been reported to survive temperatures as high as 45° C in Saudi Arabia (Aubert 1988, 1990). Heat-acclimated females might exhibit greater rates of oviposition at higher temperatures.

Female *D. citri* held at 11 °C for 48 h resumed more or less normal oviposition rates when transferred to 26° C, indicating that females are resilient to exposure to low temperatures. Whether exposure to freezes or to any temperatures below the oviposition threshold has long-term effects on reproductive output remains to be examined.

Beattie and Barkley (2009) reported that *D. citri* is better adapted to regions with high saturation deficits (high temperatures and low relative humidity levels) than to regions with low saturation deficits (medium to high temperatures and high relative humidity levels). However, reduced oviposition by *D. citri* has been reported at relative humidity levels below 40% (Skelley and Hoy 2004). Relative humidity associated with any of our temperature studies was not measured, thus the influence of humidity on upper and lower temperature thresholds of oviposition and on cold hardiness remains to be investigated.
